# Multispectral organelle imaging to model differentiation and neurodegeneration

**DOI:** 10.1002/alz70855_100362

**Published:** 2025-12-23

**Authors:** Maria Clara Zanellati

**Affiliations:** ^1^ University of North Carolina, Chapel Hill, NC, USA

## Abstract

**Background:**

Dysfunctional organelle communication networks are a common hallmark of various neurodegenerative diseases, including Alzheimer's disease (AD). Imaging organelles and their interactions in iPSC‐derived neurons that harbor disease‐associated mutations holds promise for understanding the mechanistic basis of neurodegeneration.

**Method:**

We developed a method for multispectral imaging of eight organelles simultaneously in live cells and used this method to visualize organelle morphology and dynamics (morphodynamics) along neuronal differentiation. We transfected induced pluripotent stem cells (iPSCs) and iPSC‐derived cortical neurons (iNeurons) with genetically encoded organelle markers and collected multispectral z‐stack and timelapse images at five‐time points throughout neuronal differentiation and maturation: iPSCs, and iNeurons at day 7, day 14, day 21, and day 28. Raw images were then subjected to linear unmixing and run through a Napari‐InferSubC image analysis pipeline for segmentation and analysis of approximately 1400 morpho‐metrics including organelle volume, size, shape, and number, as well as number and volume of the contacts between organelles (2‐ to 6‐way).

**Result:**

We observed dramatic remodeling of organelles during differentiation of iPSCs into iNeurons. For example, endoplasmic reticulum (ER) and mitochondria volumes increased as iPSCs differentiated into cortical neurons. We observed an increase in the overall number of the contacts throughout iNeuron maturation, accompanied by an increase in higher order contacts (3‐ and 4‐way contacts). Examples of organelle contacts that increased during iNeuron differentiation and maturation include ER‐mitochondria, known to be dysregulated in AD and Parkinson disease; mitochondria‐lysosome, previously reported defective in Charcot‐Marie‐Tooth; and ER‐peroxisome. We found that expression of VAPB, which mediates ER‐peroxisome contacts and is mutated in ALS, increases as iPSCs differentiate into iNeurons. This contact is implicated in the production of plasmalogens, which are essential for the growth and maintenance of synapses in the nervous system. Knockdown of VAPB reduced plasmalogen levels and prevented the formation of synapses during iNeuron differentiation.

**Conclusion:**

We uncovered a novel role for VAPB‐mediated ER‐peroxisome contacts in neuronal differentiation, suggesting that multi‐spectral imaging can be used to interrogate organelle morphology and contacts during neuronal differentiation and neurodegeneration. As a future direction, we will use this method to reveal defects in organelle communication networks in iNeurons with AD‐associated mutations.

